# Primary extramedullary plasmacytoma of the kidney: a case report and literature review

**DOI:** 10.3389/fonc.2026.1751965

**Published:** 2026-03-10

**Authors:** Xi Tu, Xiyao Zhuang, Fei Lai

**Affiliations:** 1 Department of Urology, West China School of Medicine, Sichuan University, Sichuan University affiliated Chengdu Second People's Hospital, Chengdu Second People's Hospital, Chengdu, Sichuan, China; 2 Department of Internal Medicine, Chengdu Shuangliu Hospital of Traditional Chinese Medicine, Chengdu, Sichuan, China

**Keywords:** extramedullary plasmacytoma, plasmacytoma, renal plasma cell tumor, renal tumor, histopathology, immunohistochemistry

## Abstract

Primary extramedullary plasmacytoma (EMP) of the kidney is a rare indolent lymphoma characterized by the monoclonal proliferation of plasma cells outside the bone marrow. Owing to its special site of origin, it is highly prone to misdiagnosis in clinical practice. Herein, we report a case of primary extramedullary plasmacytoma of the kidney. Abdominal computed tomography (CT) of the patient revealed a left renal mass, and renal carcinoma was considered. After adequate preoperative preparation, the patient underwent a radical nephrectomy for renal carcinoma. Pathological and immunohistochemical results strongly suggested the diagnosis of renal plasmacytoma.

Subsequently, the patient underwent various examinations; however, no evidence of systemic plasma cell disease was found. After the surgery, the patient refused further radiotherapy or chemotherapy. Abdominal CT was performed three months postoperatively, and no recurrence was detected. We reviewed the current case, together with previous similar reports, to further understand the characteristics, diagnosis, and treatment of this disease. Although the prognosis of EMP is relatively favorable, regular follow-up examinations are necessary because of the potential risk of recurrence or progression to plasma cell neoplasms.

## Introduction

Extramedullary plasmacytoma is a rare malignant tumor caused by clonal proliferation of atypical plasma cells. Most EMPs involve mucosal lymphoid tissues, particularly in the nasopharyngeal region, respiratory tract, and head and neck area, with approximately 80%-90% of cases occurring in the upper aerodigestive tract (1). However, EMP involving the kidneys is extremely rare. Additionally, it has been reported that approximately 11% of extramedullary plasmacytomas may progress to multiple myeloma (2). Owing to the rarity of this disease and the particularity of its anatomical location, clinical diagnosis is extremely challenging with a high misdiagnosis rate. Moreover, there are no clear treatment guidelines. Therefore, this article reports a case of primary renal extramedullary plasmacytoma and reviews the relevant literature to discuss the characteristics, diagnosis, treatment, and outcomes of renal EMP.

## Case presentation

**Figure 1:**
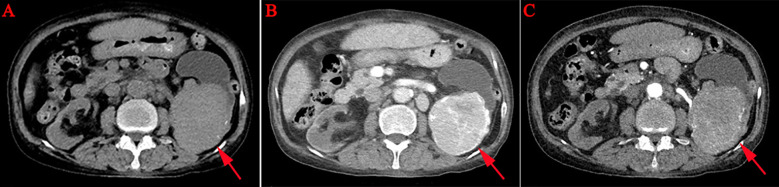
Computed tomography. These images revealed a massive soft tissue density mass in the left kidney, measuring approximately 8.5 cm × 6.4 cm, with clear boundaries, an intact capsule, and its inner edge protruding into the renal sinus, compressing the adjacent renal calyces, showing heterogeneous enhancement [(A–C), red arrow].

**Figure 2:**
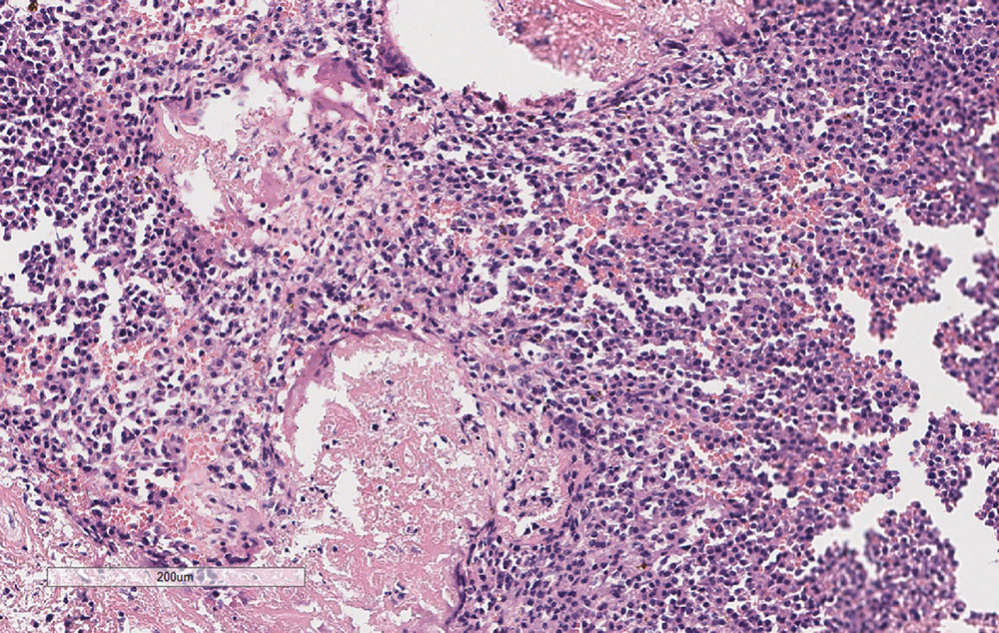
Pathology. This image revealed the tumor lesion was composed of diffuse, morphologically uniform plasma cells with atypia, and suspicious amyloid deposition was observed in the stroma (H&E, 100x).

**Figure 3:**
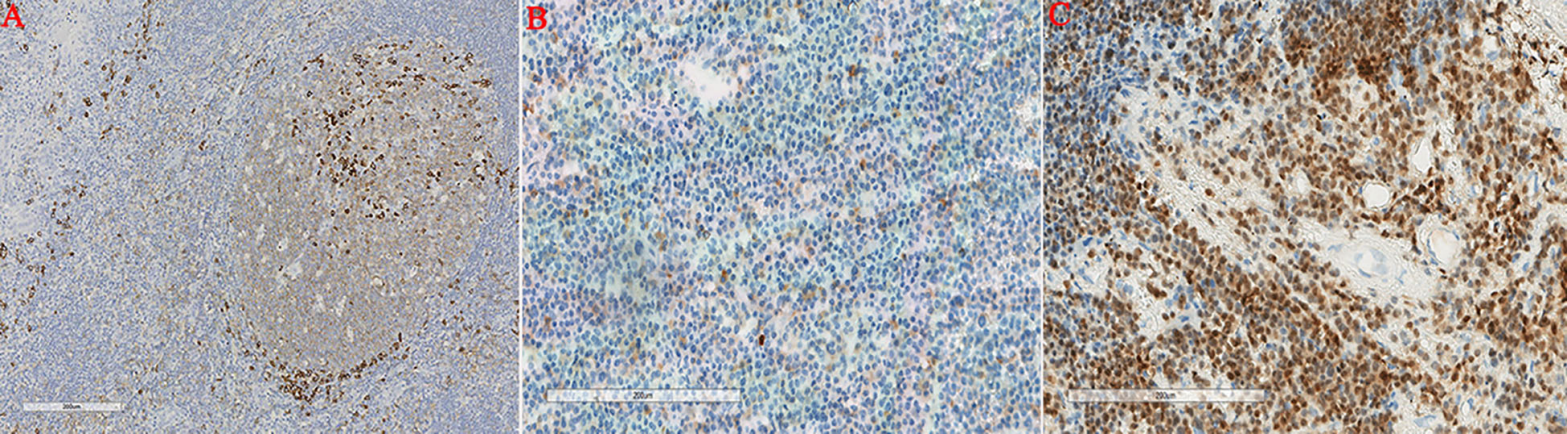
Immunohistochemical staining. Renal tumor cells showed positive CD38 expression **(A)** Renal tumor cells showed positive CD79a expression **(B)** Renal tumor cells showed positive Mum-1 expression **(C)**.

A 69-year-old female was admitted to the hospital with left lumbodorsal pain. During the course of the disease, there was no haematuria, frequent urination and pain, dizziness, palpitations, fever or chills. The patient had not received any specific treatment previously and had no family history of the disease or similar diseases. The patient’s vital signs were normal. There was no swelling, tenderness or pain induced by tapping over either kidney area. Urinalysis revealed 156 red blood cells per microliter, and biochemical test indicators were within the normal range. Abdominal CT revealed a left renal mass, and renal carcinoma was suspected (**Figure 1**). After adequate preoperative preparation, the patient underwent laparoscopic radical nephrectomy of the left kidney. Pathological examination revealed that the tumor lesion was composed of diffuse, morphologically uniform plasma cells with atypia, and suspicious amyloid deposition was observed in the stroma (**Figure 2**). Immunohistochemistry revealed that the tumor cells were positive for CD38, CD79α, Mum-1, CD138, and Ki-67, and negative for CD56, CD19, PCK, CD20, CD3, CD117, lambda, kappa, PAX-8, IgG4, and CyclinD1 (**Figure 3**). The *in situ* fluorescence hybridization test was negative for the Epstein-Barr virus. Skeletal examination showed no evidence of osteolytic lesions or active malignant tumors in other parts of the body, and bone marrow aspiration was normal. Urinalysis for the Bence-Jones protein was negative, and serum protein electrophoresis excluded multiple myeloma. No evidence of systemic plasma cell disease was found. Combined with the patient’s pathological and immunohistochemical results, a primary extramedullary plasmacytoma of the kidney was confirmed. The patient refused further therapy including external beam radiotherapy and chemotherapy. Abdominal CT performed three months post-surgery and did not reveal any relapse. The patient remained disease-free six months post-surgery. However, the patient was advised to undergo regular reexamination and lifelong follow-up.

## Systematic review of literature

PubMed, Medline, and Embase databases were systematically searched for extramedullary plasmacytoma of the kidney from 1937 to 2025. In total,93 results were obtained. After excluding articles involving secondary extramedullary plasmacytomas and cases with bone marrow involvement, 26 cases (16 men and 8 women) were finally identified, and the sex information of 2 patients could not be obtained (**Table 1**). The mean age of onset was 54.5 years (range, 14 to 76 years). The evaluation showed that 14 cases of primary extramedullary plasmacytoma of the kidney occurred in the left kidney, 8 cases in the right kidney, and 1 case in both kidneys. The main clinical manifestations of the patients were low lumbar pain, a palpable mass, and gross hematuria.

**Table 1:** Review of characteristics of primary extramedullary plasmacytoma of the kidney.

Cas no.	First author, year	Age(y)/Sex	Symptoms	Tumor location	Tumor size(cm)	SPE/IE	Bence-Jones protein	Surgery	Prognosis
1	Knudsen O, 1937 (3)	46/F	Palpable mass	NA	NA	NA	NA	Radical nephrectomy	NA
2	Farrow GM et al, 1968 (4)	53/M	NA	L	21	NA	NA	Radical nephrectomy, radiotherapy	Succumbed after 16 years
3	Siemers PT et al, 1977 (5)	56/M	Anorexia, fatigue, palpable mass	L	14	Normal	NA	Radical nephrectomy, radiotherapy, hemodialysis	Alive after 3 months
4	Silver TM et al, 1977 (6)	40/M	Gross hematuria, flank pain	L	3.5	Normal	–	Radical nephrectomy	NA
5	Kandel LB et al, 1984 (7)	55/M	Burning feeling,	R	16	γ ↑	+	Radical nephrectomy, radiotherapy	NA
6	Jaspan T et al, 1984 (8)	75/F	Back pain	L	NA	IgG ↑	+	Biopsy	Succumbed after biopsy
7	Igel TC et al, 1991 (9)	64/M	Burning feeling, weight loss	L	9	IgM ↑	NA	Radical nephrectomy, radiotherapy, chemotherapy	NA
8	Kanoh T et al, 1992 (10)	76/F	NA	NA	NA	NA	NA	Radical nephrectomy	Succumbed after 3 months
9	Shustik C et al, 1995 (11)	31/M	Asymptomatic	NA	5	IgG ↑	NA	Radical nephrectomy	Alive after 33 months
10	Manseck A et al, 1997 (12)	64/NA	NA	L	3.8	NA	–	Radical nephrectomy	NA
11	Tejido Sanchez A et al, 2001 (13)	59/NA	NA	R		NA	NA	Chemotherapy	Succumbed after 1 year
12	Fan F et al, 2005 (14)	61/F	Back pain	R	2.0	NA	NA	Partial nephrectomy, chemotherapy	Alive after 2.5 year
13	Park SY et al, 2007 (15)	39/M	NA	L	3.7	Normal	NA	Radical nephrectomy	Succumbed after 34 months
14	Yazici S et al, 2009 (16)	67/F	Asymptomatic	L	4.6	α2 ↑, γ ↑	NA	Radical nephrectomy	Alive after 6 months
15	Mongha R et al, 2010 (17)	58/M	Lumbar pain	R	9.7	Normal	–	Radical nephrectomy, radiotherapy	Alive after 1 year
16	Yang GF et al, 2010 (18)	76/F	Back pain	L	4.8	NA	NA	Radical nephrectomy	NA
17	Zhong Y et al, 2010 (19)	41/M	Epigastric discomfort	L	NA	NA	–	Radical nephrectomy	NA
18	Ozkok A, et al, 2010 (20)	68/M	NA	L	9.0	–	–	chemotherapy, radiotherapy, hemodialysis	Succumbed after 14 years
19	Zhang SQ et al, 2013 (21)	46/F	NA	L	3.8	γ ↑, α1 ↑	+	Radical nephrectomy	Alive after 6 months
20	Spence RA et al, 2013 (22)	49/M	Lumbar pain	R	NA	–	–	Radical nephrectomy	NA
21	Mei YH et al, 2017 (23)	14/F	Abdominal pain	R	3	NA	–	Radical nephrectomy	Alive after 22 months
22	Berquist SW et al, 2017 (24)	51/M	Intermittent gross haematuria	L	8	IgG	NA	Partial nephrectomy	Alive after 28 months
23	Lawrence BJ et al, 2018 (25)	69/M	chronic back pain with right-sided flank pain	R and L	NA	IgA κλ	NA	chemotherapy	Alive after 3 months
24	Li Y, et al, 2019 (26)	55/M	Abdominal pain, Gross hematuria	R	14	–	–	Radical nephrectomy, radiotherapy	Alive after 7 months
25	Niu W, et al, 2021 (27)	53/M	Frequent, painful micturition	L	12	–	–	Radical nephrectomy, Radiotherapy, chemotherapy	Succumbed after 3.5 years
26	Xia CM, et al, 2022 (28)	50/M	Lumbar pain	R	11	κ ↑, λ ↑	NA	Radical nephrectomy, Radiotherapy, chemotherapy	NA
27	Present case	69/F	Lumbar pain	L	8.5	–	–	Radical nephrectomy	–

F, female; M, male; L, left kidney; R, right kidney; NA, not available; SPE, serum protein electrophoresis; IE, immunoelectrophoresis; ↑, increased; +, positive; −, negative.

Various treatment strategies are available for primary extramedullary plasmacytoma of the kidney, including surgery, radiotherapy, chemotherapy and combination therapy. Twelve patients underwent radical nephrectomy alone (3, 6, 10–12, 15, 16, 18, 19, 21–23). Five patients underwent radical nephrectomy with radiotherapy (4, 5, 7, 17, 26). Three patients underwent radical nephrectomy combined with radiotherapy and chemotherapy (9, 27, 28). Two patients received chemotherapy alone (13, 25). Two patients underwent partial nephrectomy with or without chemotherapy (14, 24). Jaspan J et al. reported that a patient died after undergoing puncture biopsy (8). Ozkok A, et al. reported a patient who received radiotherapy and chemotherapy (20). Three cases recurred following surgery (14–16), one of which was finally diagnosed as multiple myeloma. Overall, the treatment effect was good, with a longest follow-up time of 14 years.

## Discussion

Plasma cell neoplasms are hematologic malignancies caused by the abnormal monoclonal proliferation of mature B lymphocytes, with the ability to synthesize and secrete excessive immunoglobulins and their polypeptide chain subunits (light chains or heavy chains). They are classified into four types: multiple myeloma, solitary plasmacytoma of the bone, extramedullary plasmacytoma, and plasmablastic lymphoma. EMP is a relatively rare type, with an incidence rate of only 3 per 100,000 individuals, accounting for approximately 3% of plasma cell neoplasms (29, 30). The incidence rate of extramedullary plasmacytoma in males is three times that in females, with a median age of onset of 57 years (2). EMPs are more common in the second half of life, predominantly occurring in the sixth and seventh decades of life (2). The vast majority of extramedullary plasmacytoma are most common in the head and neck region, including the nasopharynx, paranasal sinuses, and nasal cavity. They are occasionally found in the digestive and urinary systems (17, 31), whereas primary EMP occurring in the kidney is extremely rare.

The etiology and pathogenesis of extramedullary plasma cell neoplasms remain unclear, but they may be related to the following factors: ①Chronic inflammatory reactions in the kidney cause lymphocytes to infiltrate into the renal parenchyma, leading to the development of lymphoma; ②The renal capsule is rich in lymphatic vessels, and lymphocytes may develop lymphoma and infiltrate into the renal parenchyma, resulting in the disease (32).

Primary EMP of the kidney lacks specific clinical manifestations. Symptoms such as obstruction, bleeding, and pain only occur when the tumor compresses the site where it arises or when a mass can be palpated during physical examination. In the currently reported cases of renal EMP, the main reasons for seeking medical attention were hematuria, abdominal mass, low back pain, and renal insufficiency. The diagnosis of EMP is complex and usually requires radiological, hematological, biochemical, and histological examination. It is difficult to directly distinguish renal EMP from other renal tumors based solely on imaging examinations, resulting in a high misdiagnosis rate. Combined CT and computed tomography angiography (CTA) can better demonstrate invasion of the mass into the renal calyces, renal pelvis, surrounding large blood vessels, adjacent organs, and other structures. Some masses may show necrotic foci or calcifications inside (33). The final diagnosis was based on histopathological and immunohistochemical examinations. CD38 expression in the plasma cells is of great significance in the diagnosis of this disease. Other markers, such as κ or λ light chains, CD138, CD79α, and Mum-1, are also specific indicators of plasma cell origin and can be used for the diagnosis of plasmacytoma (34, 35). For further diagnosis of primary renal extramedullary plasmacytoma, the international diagnostic criteria established by the UK Myeloma Forum (UKMF) Guidelines Working Group in 2004 must be met (1): ①Pathological confirmation of an extramedullary plasma cell neoplasm, with or without regional lymph node involvement; ②Normal bone marrow examination without clonal plasma cell infiltration; ③Normal clinical and imaging examinations of the skeletal system; ④Absence of CRAB manifestations (hypercalcemia, renal insufficiency, anemia, bone lesions) caused by multiple myeloma;⑤Almost undetectable monoclonal immunoglobulin in serum or urine.

Primary EMP of the kidney is a rare malignant renal tumor. Currently, there is still a lack of sufficient clinical data on its treatment and prognosis, so no standardized treatment protocol has been established. Its treatment mainly follows the methods used for EMP, including surgery, radiotherapy, and chemotherapy, which can be used alone or in combination. A specific treatment plan can be adopted based on tumor size, clinical stage, and patient preferences. EMP is widely recognized as being highly sensitive to radiotherapy, with nearly all patients achieving local control after treatment. The optimal dose for local control is 40 to 50 Gy (depending on the tumor size) (36, 37). Radiotherapy is even recommended for patients who achieve complete remission with negative surgical margins in some studies (30, 38). Chemotherapy is an optional treatment modality for patients with refractory or recurrence disease. Different treatment methods are selected according to stage: for patients with stage I (tumor confined to the primary site) and stage II (lymph node involvement), surgical resection combined with local radiotherapy is the main approach; for patients with stage III (extensive metastasis), systemic chemotherapy is the primary treatment. Currently, the most commonly used treatment regimens are the same as those used for multiple myeloma.

It has been reported that the prognosis of EMP is closely related to tumor size, pathological grade, and clinical stage (39). Meanwhile, it is prone to recurrence, and approximately 11%% of patients may progress to multiple myeloma (2). Most of these progressions occur within 2 years of diagnosis, although there are also reports of progression to multiple myeloma 15 years after diagnosis. Therefore, long-term follow-up of patients after surgery is required.

Currently, there are few genetic studies on EMP. However, existing literature shows that the genetic abnormalities of EMP are similar to those of multiple myeloma, including DNA copy number gain (aneuploidy, polyploidy) and trisomy of chromosome 1. Additionally, abnormal plasma cells in the bone marrow of patients with EMP were detected using flow cytometry. This suggests that EMP may be an early manifestation of multiple myeloma rather than an independent tumor distinct from multiple myeloma. Nevertheless, these findings are based on small-sample studies, and further large-sample studies are required to confirm this hypothesis. Meanwhile, studies have also reported that age, tumor size, IgG λ positivity, primary lesions with adjacent bone destruction, and different treatment modalities are important factors affecting EMP transformation (40). Generally, EMP have favorable clinical outcomes and prognoses. The overall 5-year survival rate is 53%-75%, and the 10-year survival rate is higher than that of multiple myeloma (approximately 70%) (23, 41). However, the rates of *in situ* recurrence and metastasis are relatively high, reaching 30% and 40% respectively (41).

## Patient perspective

The kidney tumor brought me great trouble and anxiety, affecting my daily life. After talking to my doctor, I performed laparoscopic partial nephrectomy to remove the tumor. When histopathology and immunohistochemistry confirmed renal plasmacytoma, my concerns disappeared. Physical and psychological healing was achieved. I think I have been successfully treated. I will follow the doctors’ advice for regular follow-up in the future.

## Conclusion

Primary renal plasmacytoma is a rare disorder of plasma cells. Owing to its unusual location and lack of specific clinical symptoms, it presents diagnostic challenges. The diagnosis of EMP is complex and requires radiological, hematological, biochemical, and histological examination. A specific treatment plan can be adopted based on tumor size, clinical stage, and patient preferences. Surgery combined with radiotherapy may be the optimal treatment option. Patients with renal plasmacytoma may experience local recurrence and metastasis, and some may progress to multiple myeloma. Therefore, a long-term follow-up and close monitoring are necessary.

## Data Availability

The original contributions presented in the study are included in the article/supplementary material. Further inquiries can be directed to the corresponding author/s.
